# Supplementary figures and images for: TGF-β ligand cross-subfamily interactions in the response of Caenorhabditis elegans to a bacterial pathogen
Source: PLoS Genet. 2024 Jun 14;20(6):e1011324. doi: 10.1371/journal.pgen.1011324 (PMC11210861; doi:10.1371/journal.pgen.1011324)

*E. coli*

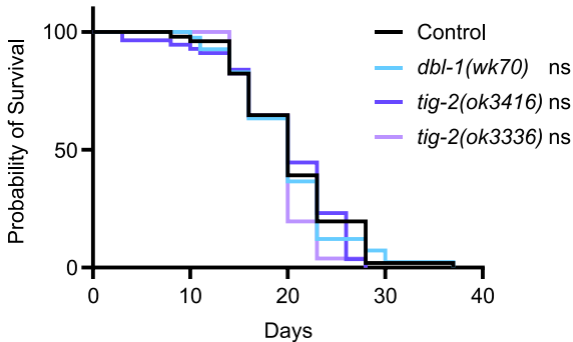

Supplement: S1 Fig — n values: Control (51), dbl-1 (41), tig-2 (56), tig-2 (51). (PDF) [file pgen.1011324.s001.pdf]

**A**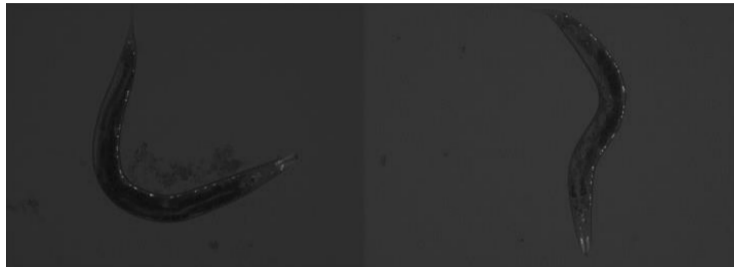

Control

Infected

**B**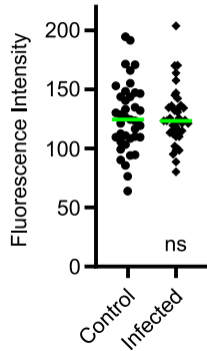

Supplement: S2 Fig — No significance. (PDF) [file pgen.1011324.s002.pdf]

**A**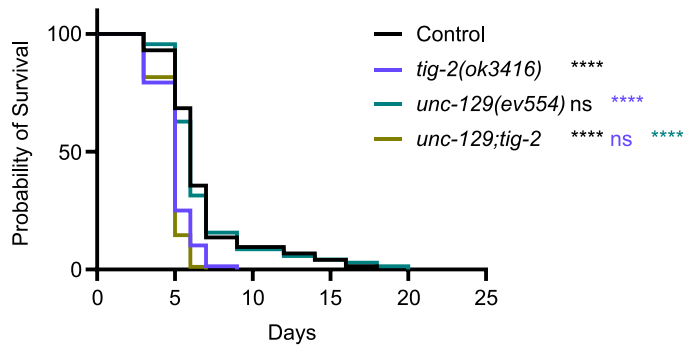**B**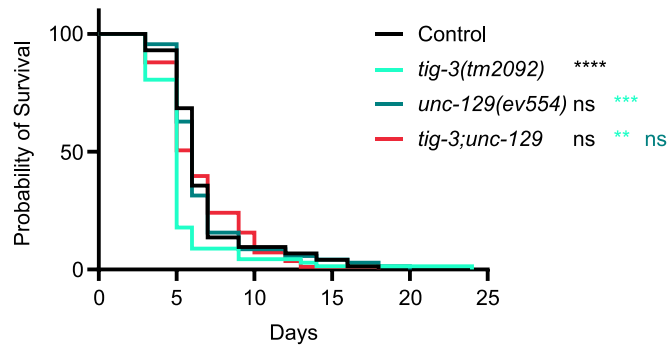**C**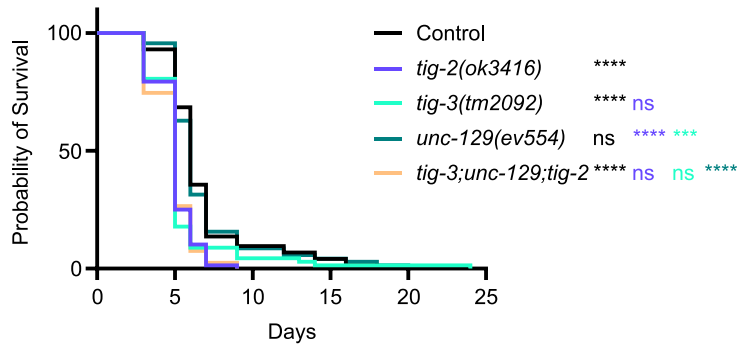

Supplement: S3 Fig — (A) n values: Control (73), tig-2 (68), unc-129 (70), unc-129;tig-2 (83). (B) n values: Control (73), tig-3 (67), unc-129 (70), tig-3;unc-129 (83). (C) n values: Control (73), tig-2 (68), tig-3 (67), unc-129 (70), tig-3;unc-129;tig-2 (79). Statistical analysis for survival analyses done using Log-rank (Mantel-Cox) test. ns p > 0.05; * p ≤ 0.05; ** p ≤ 0.01; **** p < 0.0001. Black asterisks denote significance relative to control, blue-violet is significance relative to tig-2, green-cyan is significance relative to tig-3, and teal is significance relative to unc-129. (PDF) [file pgen.1011324.s003.pdf]

■ pLDDT/100   
 ■ pTM   
 ■ ipTM  
■ BMP subfamily   
 ■ TGF- $\beta$ /Activin subfamily

**A**

Mature Ligand

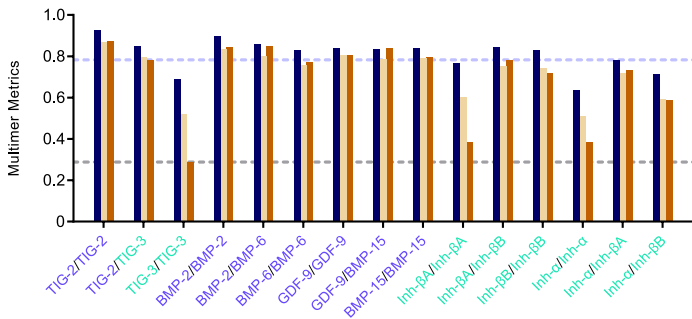

**B**

Procomplex

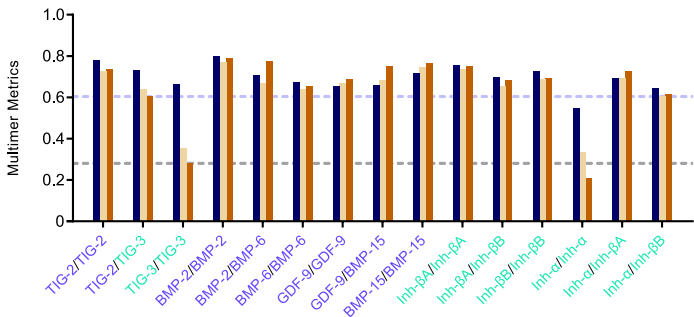

Supplement: S5 Fig — TIG-2 and TIG-3 structure predictions benchmarked against predictions for experimentally reported dimers [50,61–63] and the non-homodimeric inhibin α subunit [64]. Average pLDDT divided by 100 (navy), pTM (khaki), and ipTM (amber) metrics are grouped for each complex. Lavender and gray dashed lines correspond to TIG-2/TIG-3 and TIG-3/TIG-3 ipTM scores, respectively. Monomers are color-coded according to TGF-β subfamily, with BMP in blue-violet and TGF-β/Activin in green-cyan. Both mature and procomplexes of the TIG-2 homodimer and TIG-2/TIG-3 heterodimer converge with known dimers. In contrast, the mature and pro-forms of the TIG-3 homodimer markedly diverge with inhibin α that does not homodimerize. Mature activin A, the inhibin βA homodimer, groups with mature inhibin α despite the established ability of inhibin βA to homodimerize. Activin A procomplex, however, expectedly groups with the reported dimers. The mature and pro results together support the necessity of prodomain-mediated dimerization for activin A, as previously shown [65]. Reported non-disulfide-linked mature dimers include GDF-9/GDF-9, GDF-9/BMP-15, and BMP-15/BMP-15. Procomplex multimer metrics are observably lower (except for pTM and iPTM scores for activin A and inhibin B) than the equivalent mature form. (PDF) [file pgen.1011324.s005.pdf]
