# Supplementary material for: TGF-β ligand cross-subfamily interactions in the response of Caenorhabditis elegans to a bacterial pathogen
Source: PLoS Genet. 2024 Jun 14;20(6):e1011324. doi: 10.1371/journal.pgen.1011324 (PMC11210861; doi:10.1371/journal.pgen.1011324)
Supplement: S4 Fig — (A) Annotated TIG-2 and TIG-3 isoform B amino acid sequences. The signal sequence (black) is underlined, and the cleavage site is in boldface. The prodomain (blue-violet in TIG-2, green-cyan in TIG-3) includes the cleavage site, which separates the prodomain from the mature ligand (dark khaki in TIG-2, gray-beige in TIG-3). Cysteine residues participating in intrachain disulfide bonds are highlighted in gold. The lysine residue that replaces the dimerization cysteine is colored yellow-green. (B) Subfamily, species, Uniprot entry, signal sequence, and cleavage site for modeled TGF-β family members. (a) [60] (b) SignalP 6.0. (c) Manual sequence analysis. (d) [52]. (PDF) [file pgen.1011324.s004.pdf]

■ <u>Signal sequence</u> ■ TIG-2 prodomain ■ TIG-2 mature domain ■ TIG-3 prodomain ■ TIG-3 mature domain ■ Cys intrachain disulfide ■ Lys (replaces dimerization Cys)

A

```
>TIG-2

  1   MLFEFSILLGFLVPRGDSKGIYFVEERNGKTYTQPLTGQATDKIGEQIRE
 51   LFNIDINPNGPAVKANNYVSTYMKRLYKQLENYEHGENHNEEEVNAWLSA
101   DRIVSHMAQEVSHRLDDGSYSIRFAKEHVPAKEGQSIVRAQLRIHIQGIV
151   SPVFFYIEDTNLPGDTVLVSSDDPTVVTDVTTMVDRWSHLQLSTLPIVTA
201   RASTNDELKIEAFLVIALKDEDAGPPKKRSRRSASTTPISAPPMRQKVKR
251   SESAYFEKPNENERCQRKGLYVDFDILGWKQWVIAPEGFSAFYCSGDCSA
301   PFSKEMNATSHAIVQSTLHRVRPNSTTPAKCAPSSLGSYKILFVDQNKQV
351   QIKRYRDMVVDECGCH
```

```
>TIG-3, isoform B

  1   MHFFTRFHYCFVITIISINASSLSIERQKRQLLSALNLSSRPPLIHQAPV
 51   IPDSMSTSRKHDLYGGVLDKITLEPNRKSWTCLTPESLVKDCFQYSINSI
101   NHEILSASLIIDPKDTNISIVVYEVDELFGELQYVDRFEIRETLDKYHFD
151   ISHLFHKWMKQKSSDKMIKIEITNSNTQNVINALSVLRNAPNFDVMVFQP
201   NTVTAGTSDCVGCCVIPFYVNFTEIGWNDWILSPPGFYANVCSDTVCSTE
251   SDEVYQFMKAAISDLPEPKCAPNYYGSVDMIVALSPRDIRKTRVHGLRAL
301   SCSCT
```

B

Protein	Subfamily[a]	Species	Uniprot Entry	Signal Sequence[b]	(Prodomain	⊃ Cleavage Site)	Mature Domain
TIG-2	BMP	*C. elegans*	O16273	MLFEFSILLGFLVPRGDS	…PMRQ	KVKR[c]	SESA…
TIG-3	Activin	*C. elegans*	A0A8S4QGI4	MHFFTRFHYCFVITIISINASS	…INAL	SVLR[a]	NAPN…
BMP-2	BMP	*H. sapiens*	P12643	MVAGTRCLLALLLPQVLLGGAAG	…PLHK	REKR[d]	QAKH…
BMP-6	BMP	*H. sapiens*	P22004	MPGLGRRAQWLCWWWGLLCS	…RQQS	R[d]	NRST…
BMP-15	BMP	*H. sapiens*	O95972	MVLLSILRILFLCELVLFMEHRAQM	…ESLL	RRTR[d]	QADG…
GDF-9	BMP	*H. sapiens*	O60383	MARPNKFLLWFCCFAWLCFPISLG	…SSHH	RHRR[d]	GQET…
Inh-βA	Activin	*H. sapiens*	P08476	MPLLWLRGFLLASCWIIVRS	…HPHR	RRRR[d]	GLEC…
Inh-βB	Activin	*H. sapiens*	P09529	MDGLPGRALGAACLLLLAAGWLGPEAW	…HRIR	KR[d]	GLEC…
Inh-α	TGF-β	*H. sapiens*	P05111	MVLHLLLFLLLTPQGGHS	…SGGE	RARR[d]	STPL…
