# Supplementary material for: TGF-β ligand cross-subfamily interactions in the response of Caenorhabditis elegans to a bacterial pathogen
Source: PLoS Genet. 2024 Jun 14;20(6):e1011324. doi: 10.1371/journal.pgen.1011324 (PMC11210861; doi:10.1371/journal.pgen.1011324)
Supplement: S6 Fig — The conventional dimerization cysteine is highlighted in gold and replaced by lysine (yellow-green) in TIG-2 and TIG-3 and by serine (cyan) in GDF-9 and BMP-15. Consensus symbols (asterisk, colon, period) are defined according to standard Clustal Omega notation. (PDF) [file pgen.1011324.s006.pdf]

```
TIG-3.B          ----------NAPNFDVMVFQP-NTVTAGTSDCVGCCVIPFYVNFTEIGWNDWILSPPGF       49
INHA_HUMAN       ------STPLMSWPWSPSALRLLQRPPEEPAAHANCHRVALNISFQELGWERWIVYPPSF       54
GDF9_HUMAN       -GQETVSSELKK-PLGPASFNLSEYFRQFLLPQNECELHDFRLSFSQLKWDNWIVAPHRY       58
BMP15_HUMAN      -QADGISAEVT-----------ASSSKHSGPENNQCSLHPFQISFRQLGWDHWIIAPPFY       48
INHBA_HUMAN      -------------------------GLECDGKVNICCKKQFFVSFKDIGWNDWIIAPSGY       35
TIG-2            ---------------------SESAYFEKPNENERCQRKGLYVDFDILGWKQWVIAPEGF       39
BMP2_HUMAN       ----------------------QAKHKQRKRLKSSCKRHPLYVDFSDVGWNDWIVAPPGY       38
DBL-1            SAQTGNSERKNR--------KKGRKHHNTEAESNLCRRTDFYVDFDDLNWQDWIMAPKGY       52
                                                    *    : :.*  : *. *:: *  :

TIG-3.B          YANVCSDTVC--------ST-----ESDEVYQFMKAAISDLPEPKCA--PNYYGSVDMIV       94
INHA_HUMAN       IFHYCHGGCGLHIPPNLSLP----VPGAPPTPAQPYSLLPGAQPCCAALPGTMRPLHVRT      110
GDF9_HUMAN       NPRYCKGDCPRAVGHRYGSP----VHTMVQNIIYEKLDSSVPRPSCV--PAKYSPLSVLT      112
BMP15_HUMAN      TPNYCKGTCLRVLRDGLNSP----NHAIIQNLINQLVDQSVPRPSCV--PYKYVPISVLM      102
INHBA_HUMAN      HANYCEGECPSHIAGTSGSSLSFHSTVINHYRMRGHSPFANLKSCCV--PTKLRPMSMLY       93
TIG-2            SAFYCSGDCSAPFSKEMNAT----SHAIVQSTLHRVRPNSTTPAKCA--PSSLGSYKILF       93
BMP2_HUMAN       HAFYCHGECPFPLADHLNST----NHAIVQTLVNSVN-SKIPKACCV--PTELSAISMLY       91
DBL-1            DAYQCQGSCPNPMPAQLNAT----NHAIIQSLLHSLRPDEVPPPCCV--PTETSPLSILY      106
                     * .                                      *   *       :

TIG-3.B          ALS-PRDIRKTRVHGLRALSCSCT                                          117
INHA_HUMAN       TSDGGYSFKYETVPNLLTQHCACI                                          134
GDF9_HUMAN       IEP-DGSIAYKEYEDMIATKCTCR                                          135
BMP15_HUMAN      IEA-NGSILYKEYEGMIAESCTCR                                          125
INHBA_HUMAN      YDD-GQNIIKKDIQNMIVEECGCS                                          116
TIG-2            VDQ-NKQVQIKRYRDMVVDECGCH                                          116
BMP2_HUMAN       LDE-NEKVVLKNYQDMVVEGCGCR                                          114
DBL-1            MDV-DKVIVIREYADMRVESCGCR                                          129
                        .      .: .  * *
```
