# Supplementary material for: TGF-β ligand cross-subfamily interactions in the response of Caenorhabditis elegans to a bacterial pathogen
Source: PLoS Genet. 2024 Jun 14;20(6):e1011324. doi: 10.1371/journal.pgen.1011324 (PMC11210861; doi:10.1371/journal.pgen.1011324)
Supplement: Attachment — Submitted filename: Response to Reviewers.docx [file pgen.1011324.s007.docx]

We thank the reviewers for their constructive review of our manuscript. As detailed below, we have made extensive revisions to the text and added new experiments to address critiques. In addition, we changed the title from “Bacterial Pathogens” to “a Bacterial Pathogen” because most of the analysis was performed with *P. luminescens.* In response to specific critiques:

Reviewer #1:
*Line 133, “The larger deletion allele resulted in a survival defect similar to that of dbl-1 mutants and more pronounced than the survival defect caused by the 500 bp deletion.” Since the difference between the two TIG-2 alleles is not significantly different, stating a ‘more pronounced’ survival defect between them would be better omitted.*

**We agree with this point and deleted this sentence.** *Line 177-79, it is worth mentioning for the non-worm specialist when the dauer period is relative to L4, so the experimental scheme is more clear.*
**We added larval stage information in the Introduction at the first mention of dauer (line 92), and also here (line 179) to clarify the experimental scheme.**

*Fig 2B, it seems that the control also has a variable response to P. luminescens bacteria, when comparing it to Fig 1 panel values. The authors should comment on that. Could the P. luminescens bacteria be changing in any way that changes the survival response? Or other environmental factors that might be playing a role?*

**We have observed that the speed of killing varies between bacterial cultures. Nevertheless, the presence of a concurrent control in all experiments serves to allow interpretation of the results.** *Line 222-24, Since the double mutant has the same survival rate as the single mutants, why does that suggest they function in ‘a cooperative manner’ and what is meant by that exactly? Earlier state that it would suggest “they act together” for example as a heterodimer, when making the dbl-1 TIG double mutant. Usually one refers to acting in the same pathway (if no stronger phenotype) or in different pathways (stronger phenotype). Indeed in referring to the daf-7, dbl-1 double mutant on line 230 it is interpreted as a single pathway. Cooperativity is mentioned in that paragraph too, the abstract, and in the Discussion as well, and again is unclear. Cooperative protein interactions mean something completely different, hence, this terminology in this context is a bit confusing.*

**We agree that “cooperative” can have a meaning that is very different from our intention. We therefore removed this term from the paper. Instead, we use the more neutral wording “act together” or “nonredundantly”.** *Fig 3B, shouldn’t the daf-7 mutant alone be shown too for comparison?*

**We performed additional simultaneous trials to compare *daf-7;dbl-1* with both single mutants. This experiment is now shown in Figure 3C.** *Line 242, a typo. One of those should be TIG-3.*
**Thank you, we corrected this typo.**

*Figure 4, might there be some functional overlap or redundancy between the Type I receptors and R-Smads.*

**There could be some redundancy between the sets of components. We added a caveat to this effect (lines 283-285).** *Figure 7A, right panel, the “Interchain Residue Contacts” label is located in panel C, better if moved up into panel A region.*

**This label describes the data in panel C, not panel A.** *Discussion
Line 431: “Here we demonstrate that all five TGF-β ligands play a role in the immune response
against bacterial pathogen”. All 5 ligands have been demonstrated to play a role in survival to pathogenic bacteria, does that necessarily mean they play a role in the immune response? The results presented seem insufficient to state that. Either further evidence should be provided or these statements modified.*
**We removed the term “immune response” and replaced it with the broader “survival”.**

*Zhang and Zhang, 2012, and Zugasti and Ewbank, 2009, are missing from the Reference list.*

**We added these references to the reference list.**

Reviewer #2:
*1- All five TGF-β ligands play a role in the immune response – the authors demonstrate immune response solely based on survival studies.*

**We changed “immune response” to “survival” (line 32).**

*2- The immune response is not a general one-size-fits-all response to bacterial pathogens but rather a more nuanced response with some level of pathogen specificity. Why only two-gram negative bacteria were used?*

**While we certainly expect that new information can be gained by testing additional bacteria, the matrix of possible components and possible bacteria expands quite quickly and would be beyond the scope of a single publication.**

*3- The authors looked for interactions between the C. elegans TGF-β ligands upon exposure to bacterial infection by testing double mutants. DAF-7 and TIG-3 are classified as TGF-β/Activin-like ligands, whereas DBL-1 and TIG-2 are BMP ligands. What is the rationale for the combinations used? For example, why TIG-3/DBL-1 was not studied?*

**We did not previously have the *tig-3;dbl-1* double mutant. To address this comment, we generated the double mutant and analyzed its survival on *P. luminescens* (Figure 3B and page 13). Our results are consistent with the conclusion DBL-1 and TIG-3 act independently.**

*4- The survival of dbl-1 and unc-129 animals show variability, which questions the reproducibility of the data and the reliability of the involvement of these ligands in defense (Fig 2 A, B).*

**Survival defects for *dbl-1* mutants and for *unc-129* mutants were observed in multiple trials, but the magnitude of the effect is somewhat variable. In particular, the faster lethality on *P. luminenscens* narrows the difference between mutants and control animals. In the trial shown in Figure 2B, the survival deficit of *dbl-1* mutants does not achieve the level of statistical significance. We believe that an important conclusion from these experiments is that TIG-2 and TIG-3 play a more critical role in survival on *P. luminescens* than DBL-1 or UNC-129.**

*5- Figure 3B lacks daf-7(m62) single animals. “Survival of daf-7;dbl-1 double mutants was not significantly different compared to dbl-1 single mutants (Line 228)”. However, there is difference between dbl-1(wa70) and daf-7(m62) animals in Fig 2A. Is there any difference between daf-7(m62) animals and double mutant animals?*

**We performed additional simultaneous trials to compare *daf-7;dbl-1* with both single mutants. This experiment is now shown in Figure 3C.**

*6- How many animals were used per trial, and how many biological and technical replicates were used in the study?*

**All figure legends show the n values. All experiments were performed with at least two, and usually three, biologically independent replicates.**

*Minor
Low contrast curves were used in survival figures Fig S3.*

**We have uploaded a new version of Fig S3.**

*Lacks reference at line 217.*

**We added the reference to Figure 1C.**

*Line 237, a typo, Fig S3 rather Fig S1.*

**Thank you, we fixed these references.**

*Fig S6 was mentioned ahead of Fig S4. Fig S5 was never mentioned.*

**We renumbered the supplemental figures and added the missing reference.**

Reviewer #3:
*1) The authors showed that tig-2 dbl-1 double mutants exhibited a more severe phenotype than either single mutants in their response to P. luminescens, while tig-3; tig-2 double mutants behaved similarly to either tig-3 or tig-2 single mutants in their responses to P. luminescens. They concluded that TIG-2 and DBL-1 have additive, while TIG-2 and TIG-3 have cooperative roles, in the immune response. What are the phenotypes of tig-3; dbl-1 double mutants or tig-3; tig-2 dbl-1 triple mutants? Results from these analyses will help clarify the relationship among these three ligands in the immune response. The reason is the following: the authors first showed in Figure 1 that tig-2 dbl-1 double mutants exhibited a much more severe phenotype than either tig-2 or dbl-1 single mutants, but then showed in Figure 5 that sma-3 mutants behaved similarly to either tig-3 or tig-2 single mutants, but exhibited a more severe phenotype than dbl-1 single mutants. If as the authors argued that TIG-2 and TIG-3 function through the canonical BMP pathway mediated by SMA-3, does DBL-1 then function via a non-canonical BMP pathway?*

**We did not previously have the *tig-3;dbl-1* double mutant. To address this comment, we generated the double mutant and analyzed its survival on *P. luminescens* (Figure 3B and page 13). Our results are consistent with the conclusion DBL-1 and TIG-3 act independently as expected.**

**The question about SMA-3 function is an excellent one. We hypothesize that DBL-1 acts via SMA-3, because *sma-3* mutants and *dbl-1* mutants have the same failure to induce antimicrobial peptide genes *abf-2* and *cnc-2* in response to *P. luminescens* (Ciccarelli et al., 2024, MBoC). At this time,** **we do not know why the *sma-3* survival defect is not worse than that of *tig-2* or *tig-3* mutants. It is possible that activation of SMA-2 in a *sma-3* mutant background partially compensates for the loss of *sma-3* and that this activation is lacking in *tig-2dbl-1* and *tig-3;dbl-1* double mutants causing a more severe phenotype. We added these considerations to the Discussion (lines 480-488).**

*2) It would be helpful to show in a supplemental Figure where the deletions are for the two tig-2 deletions alleles and how they may impact on the molecular structure of TIG-2.*

**These alleles have not been curated, so we know size of the deletions based on PCR assays, but not there locations.**

*3) It is unclear why there is no error bar for the control samples in the qRT-PCR results shown in Figure 2.*

**Relative expression is normalized to the level in the control samples, so the control always has a value of 1.0 by default.**

*4) Please comment on why the control animals for sma-4 mutants exhibit a much shorter life span than other control animals in Figure 4.*

**We have observed that the speed of killing varies between bacterial cultures. Nevertheless, the presence of a concurrent control in all experiments serves to allow interpretation of the results.** *Minor:
1) Line 77, Zamarron and Chen, 2011 (rather than 20011)
2) Line 430, “in the context of an intact organism” (rather than “the”)
3) Figure S3 legend: unc-129(ev554); tig-2(ok3416) (rather than unc-129(ev554);tig-2(3416))*

**We thank you for catching these typos. We have made the corrections.**
